# Effects of yeast peptides on the growth, antioxidant and anti-inflammatory capacities, immune function, and diarrhea status of suckling calves

**DOI:** 10.3389/fvets.2024.1454839

**Published:** 2024-10-10

**Authors:** Xuexian Liu, Jiashu Yang, Yibo Yan, Kai Wang, Chunyan Guo

**Affiliations:** ^1^Department of Bioengineering, Jinzhong Vocational and Technical College, Jinzhong, China; ^2^College of Veterinary Medicine, Shanxi Agricultural University, Jinzhong, China; ^3^College of Animal Science, Shanxi Agricultural University, Jinzhong, China

**Keywords:** yeast peptide, calves, diarrhea, suckling period, immune function

## Abstract

Yeast peptides, which are small-molecule active peptides extracted from yeast proteins, are known for their antibacterial, antioxidant, and anti-inflammatory properties. However, the effects of yeast peptide on suckling calves remain unclear. In this study, the effects of yeast peptide supplementation on the growth, diarrhea incidence, and immune function of calves during the suckling period were determined. Thirty newborn calves were randomly divided into two groups: the control group (CON) and the treatment group (AP), which received fresh pasteurized milk supplemented with yeast peptides (5 g/day). The experiment lasted for 49 days (7–56 days of age). The dry matter intake, body weight, diarrhea status, immune function, antioxidant capacity, and anti-inflammatory activity of the calves were analyzed. The AP group had higher dry matter intake, daily weight gain, and feed efficiency than the CON group (*P* < 0.05). Moreover, the duration and frequency of diarrhea were significantly lower in the AP group than in the CON group (*P* < 0.05). Furthermore, the immune, antioxidant, and anti-inflammatory capabilities of the AP group were significantly higher than those of the CON group (*P* < 0.05). These findings provide valuable insights for the improvement of early health management during calf rearing.

## 1 Introduction

Calf rearing during the lactation period is a significant challenge in large-scale dairy farms (1). Diarrhea in newborn calves is the primary reason for calf culling and death, resulting in reduced average daily weight gain of calves within 6 months and increased annual breeding cost of ~$36 per heifer (2–4). The morbidity rate of calves during the suckling period is ~34%; meanwhile, the mortality rate is ~5%, with diarrhea accounting for 53–63% of these deaths (3). Thus, further research is required to promote the healthy growth of calves and reduce the economic losses due to calf mortality caused by early breeding.

Yeast peptides are small-molecule active peptides extracted from yeast proteins, primarily produced through the fermentation systems of yeast strains with various biological activities and nutritional value, such as brewing yeast and *Saccharomyces cerevisiae* (5). The main components of yeast peptides are amino acids, including essential and non-essential amino acids required by humans and animals (6). Yeast peptides are efficient sources of protein because they are easy to digest and absorb. Aside from being inexpensive, yeast peptides do not produce residues and exhibit a short cultivation cycle, strong antibacterial activity, and resistance to drugs, heat, acids, alkalis, and enzymes (7, 8). In an effort of reducing the application of antibiotics in China's animal breeding industry (9), yeast peptides are often used as feed additives in breeding animals (10).

Recent studies in animal models have shown that yeast peptides play an important role in regulating intestinal flora (11–13) by promoting the growth of beneficial bacteria (such as *Bifidobacterium* and *Lactobacillus*) while inhibiting the proliferation of harmful bacteria (such as *Escherichia coli* and *Salmonella*) (11–13), leading to improved intestinal health and enhanced immunity (13). Moreover, yeast peptides exhibit antioxidant and anti-inflammatory properties (14, 15). Yeast peptides alleviate oxidative stress and protect cells from oxidative damage by scavenging free radicals and increasing the activity of antioxidant enzymes (16, 17). Yeast peptides induce anti-inflammatory effects by regulating the production and release of various inflammatory factors (16, 17). Li et al. (18) found that yeast peptides can effectively alleviate weaning stress, increase feed intake, enhance immunity, and improve the intestinal mucosal structure in ruminants. However, the feasibility of administering yeast peptides to calves during the suckling period has not yet been explored.

Hence, this study was conducted to determine the effects of yeast peptide supplementation on the growth performance, immune function, and health of Holstein calves during the suckling period. We hypothesized that yeast peptide supplementation could effectively alleviate diarrhea and improve the growth performance and immune, antioxidant, and anti-inflammatory abilities of Holstein calves. The findings of this study contribute to the development early-breeding strategies for calf rearing.

## 2 Materials and methods

### 2.1 Animals, diet, and experimental design

The study was conducted in October 2021 at a commercial farm in Shanxi Province, China. Thirty newborn bull calves were included in this study. Each calf was individually housed in a calf hutch (1.5 × 3 × 1.2 m3) with rice husk bedding. The calf hutches were cleaned daily before 07:00; the bedding was replaced and lime powder was spread throughout to ensure the health and hygiene of the calves. All calves were fed the same diet; each calf received 6 L of colostrum within 1 h of birth, followed by an additional 2 L of the same colostrum 6 h later to establish passive immunity. Subsequently, the calves were provided with fresh pasteurized milk twice daily (at 08:00 and 18:00) using a milk pail. From birth to 28 days of age, the calves were fed 10 L of milk; however, from 28 to 56 days of age, they were fed 13 L of milk. Starting from the third day of postpartum, the calves were provided *ad libitum* access to commercial starter feed (water content: 11.34%; crude protein: 23.4%; crude ash: 7.22%; crude fiber: 8.3%; calcium: 1.12%; phosphorus: 0.61%; without any additives or plant extracts; Lian Ying, Tianjing, China) and fresh clean water at 39°C.

The 30 calves (40.69 ± 1.09 kg) were randomly assigned to two treatment groups: the control group (CON) and the yeast peptide (AP) feeding group. From Day 7 until the end of the experiment (at 56 days of age), the AP group was fed daily with milk (2.5 g per feed) supplemented with 5 g of yeast peptide. The dosage of 5,000 mg/kg was based on a previously published article on yeast peptide supplementation in sheep (18). The yeast peptide provided by Enhalor International, Beijing, China, is a 19-amino-acid-long antimicrobial peptide (GGVGKIIEYFIGGGVGRYG) produced via yeast fermentation. It is composed of crude protein (≥3%) and mannan (0.5%) and concentration is 5,000 mg/kg.

### 2.2 Measurement of feed intake and growth performance

At birth, 28 days, and 56 days of age, the body weights of the calves were measured before the morning feeding using a digital scale with a range of 0–200 kg. Additionally, the experiment staff recorded the feed intake of starter feed and milk at 0820 daily to calculate the average dry matter intake (DMI) of the calves. The average daily gain (ADG) and feed efficiency of the calves were calculated every 28 days. Feed efficiency was calculated as ADG/DMI.

### 2.3 Blood collection and measurement

At 28 days and 56 days of age, blood samples were collected from the jugular vein of all calves 2 h after the morning feeding using 10 ml EDTA-containing tubes to measure immune, inflammatory, and antioxidant indices in the blood. The collected blood was centrifuged at 3,000 ×*g* at 4°C for 20 min to obtain plasma, which was then stored in 1.5 ml centrifuge tubes and placed in liquid nitrogen. The concentrations of serum immunoglobulin (Ig) G, IgA, IgM, interleukin-6 (IL-6), IL-8, and IL-10 were determined using respective ELISA kits (Jinhaikeyu Biological Technology Development Co. Ltd., Beijing, China). Total antioxidant capacity (T-AOC), superoxide dismutase (SOD), malondialdehyde (MDA), and catalase (CAT) activity in the serum were de-termined using commercial kits (Nanjing Jian Cheng Bioengineering Institute, Nanjing, China).

### 2.4 Diarrhea scoring and medication

Between 0830 and 1,100 h daily, one experienced veterinarian assessed the fresh feces of all calves using a fecal scoring system. The veterinarian was blinded to the group allocation of the calves to ensure the objectivity and accuracy of the assessment. The fecal scoring ranged from 1 to 4, as referenced by Renaud et al. (19), with the following definitions: fecal score 1 = normal consistency; 2 = semi-formed or pasty; 3 = loose feces; and 4 = watery feces. Diarrhea was defined as a fecal score of ≥3 for at least two consecutive days. Referring to the method of Hu et al. (20), calves diagnosed with diarrhea were treated with 2 L/d electrolyte solution (containing 118 mg sodium chloride, 745 mg glucose, 85 mg sodium bicarbonate, and 50 mg potassium chloride per g), administered between milk feedings until the fecal score was ≤2. Timely supplementation with electrolytes prevented dehydration, which could otherwise lead to mortality or severe illness, thereby impacting the accuracy of the experiment. Additionally, throughout the entire trial, veterinarian did not observe any other types of diseases in the calves, such as pneumonia.

### 2.5 Statistical analysis

All statistical analyses in this study were performed using R software (v. 4.0.3). The normality of all data was tested using the shapiro function in R software (v. 4.0.3), and data that were not normally distributed were log-transformed before analysis. Next, data from a single age point for indicators of feed performance, growth performance, serum immunity, serum inflammation, and serum antioxidants were analyzed via a mixed linear model, the relevant model as follows:


(1) $$\begin{array}{l} Y_{ijk}=\mu +T_{i}+Calf_{j}+e_{ijk} \end{array}$$

where *Y*_*ijk*_ is the dependent variable; μ is the average experimental value; *T*_*i*_ is the fixed effect of AP supplementation or not; *Calf*_*j*_ is the random effect of calf; *e*_*ijk*_ is the error term. In addition, data form the whole periods for these indicators were analyzed via a mixed linear model, the relevant model as follows:


(2) $$\begin{array}{l} Y_{ijkl}=\mu +T_{i}+D_{j}+TD_{ij}+Calf_{k}+e_{ijkl} \end{array}$$

where *Y*_*ijkl*_ is the dependent variable; μ is the average experimental value; *T*_*i*_ is the fixed effect of AP supplementation or not; *D*_*j*_ designates the repeated (day) effect; *TD*_*ij*_ is the interaction effect of *T*_*i*_ and *D*_*j*_; *Calf*_*k*_ is the random effect of calf; *e*_*ijkl*_ is the error term. The incidence of diarrhea and medication during the experiment was analyzed using logistic regression for binomial distribution with the family=binomial function of glm function in R software (v. 4.0.3). Odds ratios were used to compare the likelihood of calves experiencing any event during each treatment. The duration of diarrhea and the number of medication days were evaluated using Poisson regression with the family=binomial function of glm function in R software (v. 4.0.3). A *P* < 0.05 was considered statistically significant, and 0.05 ≤ *P* < 0.10 was considered a tendency.

## 3 Results

### 3.1 Diarrhea and medication

Table 1 shows the effects of AP treatment on the health status in the suckling calves. Throughout the experiment, the duration of diarrhea was significantly longer in the CON group than in the AP group (14.59 vs. 10.96, *P* < 0.01). Similarly, the number of days requiring medication for diarrhea was significantly higher in the CON group than in the AP group (5.93 vs. 3.44, *P* < 0.01). Moreover, the probability of diarrhea occurrence was higher in the CON group than in the AP group (OR = 3.14, *P* < 0.01). Furthermore, the incidence of diarrhea requiring medication was higher in the CON group than in the AP group (OR = 2.36, *P* < 0.01).

**Table 1:** Effect of yeast peptide supplementation on the health status of suckling Holstein calves.

**Items**	**Treatment** ^**a**^		**SEM**	***P*-value**	**OR^b^**	**coefficient**
	**CON**	**AP**				
Diarrhea (≥3), d	14.59	10.97	0.10	0.006		
Medication (for diarrhea), d	5.93	3.44	0.18	0.002		
Diarrhea occurrence (CON VS AP)			0.12	0.002	3.14	0.29
Diarrhea medication occurrence (CON VS AP)			0.12	0.002	2.36	0.86

^a^CON, milk without yeast peptides; AP, milk supplemented with yeast peptides (5 g/d per calf).

^b^The odds ratio (OR) indicates the probability of having diarrhea (≥3) or medication. An OR > 1 indicates that the CON treatment is a risk factor for developing diarrhea compared with AP treatment.

### 3.2 Feed intake and growth performance

Table 2 shows the effects of AP treatment on the body weight, ADG, starter feed DMI, total DMI, and feed efficiency of the calves during the suckling period. There was no significant difference in the initial body weight of calves between the CON and AP groups (*P* = 0.433). However, at 28 and 56 days of age, the body weight of the calves in the AP group was significantly higher than that of the calves in the CON group (*P* < 0.01). Additionally, the ADG, starter DMI, total DMI, and feed efficiency of the calves exhibited significant differences in treatment effects and interaction effects of treatment and age (*P* < 0.01). Therefore, the treatment method had a significant impact on the growth performance of the calves, and its effects varied significantly with age.

**Table 2:** Effect of yeast peptide supplementation on the feed intake and growth performance of suckling Holstein calves.

**Items**	**Treatment** ^**a**^			***P*** **-value** ^**b**^		
	**CON**	**AP**	**SEM**	**T**	**A**	**T** ^*^ **A**
**Body weight, kg**						
0 d	40.53	40.85	0.20	0.433		
28 d	57.63	61.28	0.41	<0.01		
56 d	91.84	95.74	0.49	<0.01		
ADG, kg/d	0.95	1.02	0.01	<0.01	<0.01	<0.01
Starter DMI, kg/d	0.78	0.93	0.02	<0.01	<0.01	<0.01
Total DMI^c^, kg/d	2.20	2.35	0.02	<0.01	<0.01	<0.01
Feed efficiency^d^	2.32	2.29	0.001	<0.01	<0.01	<0.01

^a^CON, milk without yeast peptides; AP, milk supplemented with yeast peptides (5 g/d per calf).

^b^T, treatment effect; A, age effect; T^*^A, interaction between treatment and age.

^c^Total DMI = starter DMI + milk DMI.

^d^Feed efficiency = total DMI/ADG.

DMI, dry matter intake; ADG, average daily gain.

### 3.3 Serum immunoglobulin concentrations

Table 3 shows the effects of AP treatment on the serum immunoglobulin (IgG, IgM, and IgA) levels in the suckling calves. The IgG concentration in AP calves significantly increased at 28 days of age and throughout the growth period (*P* < 0.01); however, the interaction effect of treatment and age on IgG concentration was not significant (*P* = 0.284). At 28 and 56 days of age and throughout the growth period, both the treatment effect and interaction effect of treatment and age on IgM concentration were not significant (*P* = 0.561). Similarly, the effect of treatment on serum IgA concentration in calves was not significant at 28 and 56 days of age (*P* = 0.164 and *P* = 0.197, respectively). However, throughout the experimental period, the IgA concentration in the AP group tended to be higher than that in the CON group (*P* = 0.065); however, the interaction effect of treatment and age on IgA concentration was not significant (*P* = 0.725).

**Table 3:** Effect of yeast peptide supplementation on the serum immunoglobulin levels in suckling Holstein calves.

**Items**	**Treatment** ^**a**^			***P*** **-value** ^**b**^		
	**CON**	**AP**	**SEM**	**T**	**A**	**T** ^*^ **A**
**IgG, g/L**						
28 d	4.13	5.24	0.99	<0.01		
56 d	5.33	5.87	1.21	0.233		
Overall	4.73	5.55	1.07	<0.01	<0.01	0.284
**IgM, g/L**						
28 d	2.40	2.55	0.26	0.131		
56 d	2.66	2.73	0.29	0.572		
Overall	2.53	2.64	0.27	0.153	<0.01	0.561
**IgA, g/L**						
28 d	0.70	0.74	0.07	0.164		
56 d	0.76	0.81	0.12	0.197		
Overall	0.73	0.77	0.09	0.065	<0.01	0.725

^a^CON, milk without yeast peptides; AP, milk supplemented with yeast peptides (5 g/d per calf).

^b^T, treatment effect; A, age effect; T^*^A, interaction between treatment and age.

IgG, immunoglobulin G; IgM, immunoglobulin M; IgA, immunoglobulin A.

### 3.4 Serum anti-inflammatory capacity

Table 4 shows the effects of AP treatment on the serum inflammatory cytokine levels (TNF-α, IL-6, IL-8, and IL-10) in the suckling calves. There was no significant difference in serum TNF-α concentrations between the CON and AP groups at 28 and 56 days of age and throughout the growth period (*P* > 0.05), indicating that the treatment effect was not significant. The IL-6 and IL-8 concentrations in the serum of the AP calves significantly decreased at 28 days of age and throughout the growth period (*P* < 0.01), and the interaction effect of treatment and age was significant (*P* < 0.01); however, there were no significant differences in these indicators at 56 days of age. Similarly, the IL-10 concentration in the serum of the AP calves significantly increased at 28 days of age and overall growth period, and the interaction effect of treatment and age was significant (*P* < 0.01); however, there were no significant differences in these indicators at 56 days of age. Therefore, AP treatment had a significant effect on IL-6, IL-8, and IL-10 concentrations.

**Table 4:** Effect of yeast peptide supplementation on serum cytokine levels in pre-weaned Holstein calves.

**Items**	**Treatment** ^**a**^			***P*** **-value** ^**b**^		
	**CON**	**AP**	**SEM**	**T**	**A**	**T** ^*^ **A**
**TNF-**α**, pg/mL**						
28 d	46.99	49.62	1.03	0.208		
56 d	20.03	19.45	0.63	0.660		
Overall	33.51	34.54	0.80	0.397	<0.01	0.190
**IL-6, pg/mL**						
28 d	91.91	84.40	1.02	<0.01		
56 d	78.73	77.75	0.60	0.421		
Overall	85.32	81.07	0.78	<0.01	<0.01	<0.01
**IL-8, pg/mL**						
28 d	63.93	50.20	1.57	<0.01		
56 d	40.96	41.16	0.41	0.813		
Overall	52.45	45.68	0.91	<0.01	<0.01	<0.01
**IL-10, pg/mL**						
28 d	17.22	19.36	0.24	<0.01		
56 d	19.56	19.41	0.14	0.581		
Overall	18.39	19.38	0.16	<0.01	<0.01	<0.01

^a^CON, milk without yeast peptides; AP, milk supplemented with yeast peptides (5 g/d per calf).

^b^T, treatment effect; A, age effect; T^*^A, interaction between treatment and age.

TNF-α, tumor necrosis factor- α; IL-6, interleukin-6; IL-8, interleukin-8; IL-10, interleukin-10.

### 3.5 Serum antioxidant capacity

Table 5 shows the effects of AP treatment on the serum antioxidant (MAD, T-AOC, SOD, and CAT) levels in the suckling calves (Table 5). The MAD concentration did not significantly differ between the CON and AP groups at 28 and 56 days of age (*P* > 0.05), and there were no significant differences in the overall treatment effect and the inter-action between treatment and age (*P* > 0.05). Similarly, the SOD and CAT concentrations were not significantly affected by treatment at 28 and 56 days of age (*P* > 0.05), and there were no significant differences in the overall treatment effect or the interaction between treatment and age (*P* > 0.05). Additionally, the T-AOC values in the AP group were significantly lower than those in the control group at 28 days of age (*P* < 0.05); however, there was no significant difference at 56 days of age (*P* > 0.05). Although the overall treatment effect on T-AOC concentration was not significant, the interaction between treatment and age significantly influenced the T-AOC concentration in calves.

**Table 5:** Effect of yeast peptide supplementation on the serum antioxidant status of pre-weaned Holstein calves.

**Items**	**Treatment** ^**a**^			***P*** **-value** ^**b**^		
	**CON**	**AP**	**SEM**	**T**	**A**	**T** ^*^ **A**
**MAD, nmol/mL**						
28 d	3.56	3.35	0.06	0.094		
56 d	3.19	3.22	0.06	0.832		
Overall	3.38	3.29	0.06	0.318	<0.01	0.191
**T-AOC, U/mL**						
28 d	21.96	16.73	1.19	0.025		
56 d	21.59	25.40	1.24	0.126		
Overall	21.77	21.07	1.14	0.669	0.014	<0.01
**SOD, U/mL**						
28 d	87.29	87.95	1.41	0.684		
56 d	83.07	82.54	1.92	0.893		
Overall	84.66	85.25	1.69	0.893	0.066	0.726
**CAT, U/mL**						
28 d	36.10	36.14	1.12	0.988		
56 d	44.46	46.44	1.31	0.458		
Overall	40.28	41.29	1.21	0.565	<0.01	0.579

^a^CON, milk without yeast peptides; AP, milk supplemented with yeast peptides (5 g/d per calf).

^b^T, treatment effect; A, age effect; T^*^A, interaction between treatment and age.

MAD, malondialdehyde; T-AOC, total antioxidant capacity; SOD, superoxide dismutase; CAT, catalase.

## 4 Discussion

Yeast peptides are protein fragments extracted from yeast strains that are typically rich in amino acids, peptide chains, and bioactive substances. Currently, yeast peptides have been proven as feed additives that exhibit anti-stress functions, promote animal growth, and regulate immunity. However, studies on yeast peptide supplementation in calves are limited. During the suckling period, the gastrointestinal physiology of calves develops and maybe subjected to diseases such as diarrhea. To the best of our knowledge, this is the first study to report the effects of yeast peptides on suckling calves. The results revealed that yeast peptide supplementation alleviated diarrhea in suckling calves and enhanced their feed intake, immunity, and antioxidant and anti-inflammatory capabilities.

Health is a key factor that affects the growth performance of calves. Compared with the control treatment, yeast peptide supplementation significantly reduced the duration and incidence of diarrhea as well as the frequency of medication. A previous study reported that yeast culture, which includes yeast cells and their metabolic products, can improve calf fecal scores, reduce the number of days with watery feces, and decrease the incidence of diarrhea (21, 22). As a component of yeast culture, yeast peptides may also play a key role in alleviating calf diarrhea. The short-chain amino acids and small peptides in yeast peptides provide nutrition for beneficial microorganisms, including lactic acid bacteria and bifidobacteria, thereby promoting their growth and reproduction (22). By promoting the growth of beneficial bacteria, yeast peptides can inhibit the proliferation of harmful pathogens, improve the balance of gut microbiota, and increase the production of short-chain fatty acids, thereby alleviating calf diarrhea (23, 24). Due to their direct antimicrobial activity, yeast peptides can inhibit the growth of various pathogens and reduce their invasion of the intestine (25, 26). It has also been reported that yeast peptides can alleviate intestinal damage in weaned lambs (18), which may also be the mechanism for alleviating calf diarrhea, as evidenced by the significantly improved average daily gain and feed efficiency of the calves examined in this study. As dehydration, loss of appetite, and nutrient absorption disorders caused by diarrhea directly reduce the growth rate of calves (27), the higher growth performance of the AP calves may be due to the shorter duration and lower frequency of diarrhea. Although this study did not focus on the digestibility of nutrients in calves, previous studies have shown that yeast cultures can improve nutrient digestibility in calves (28). Thus, higher nutrient digestibility may explain the improved growth performance of the AP calves.

Immunoglobulins are glycoproteins produced by B cells that proliferate and differentiate into plasma cells upon antigen stimulation and are present in the serum (29). The serum concentrations of IgG, IgA, and IgM reflect the immunity of animals. The immune system of suckling calves is not well-developed and relies primarily on passively acquired maternal immunoglobulins, especially IgG (1, 30). In this study, compared with the control treatment, yeast peptide supplementation significantly enhanced the immunity of suckling calves, which was mainly reflected in the elevated concentrations of serum IgG and IgA throughout the experiment. Yeast peptides are abundant in amino acids that facilitate protein synthesis and immune cell function, support normal immune system function, increase immune cell vitality and quantity, and promote antibody production (such as IgG and IgA). IgM is typically associated with the initial response to infection. The lack of significant differences in IgM levels throughout the trial period may also indicate that the experimental calves were not infected by viruses or bacteria at 28 and 56 days after birth. Previous studies have highlighted that yeast peptides can improve the gut health of calves by promoting the proliferation of beneficial bacteria while inhibiting the growth of harmful bacteria. The optimization of the gut microbiota can enhance the function of the intestinal immune system, increasing both local and systemic immune responses, and thereby increasing IgA and IgG concentrations. In summary, yeast peptide supplementation increases the serum immunoglobulin levels in suckling calves. However, the mechanisms underlying the effects of yeast peptides on calf immunity remain unclear, necessitating further research.

Cytokines, including pro-inflammatory factors (such as TNF-α, IL-6, and IL-8) and anti-inflammatory factors (such as IL-10) (31), play important roles in initiating, regulating, and terminating inflammatory responses, protecting the body from infections and damage while preventing tissue damage caused by excessive inflammation (31, 32). In this study, yeast peptides significantly reduced the concentration of pro-inflammatory factors (IL-6 and IL-8) in the calf serum and increased the concentration of anti-inflammatory factors (IL-10). Typically, under disease or stress stimuli, the concentrations of pro-inflammatory factors in animal serum increase, whereas anti-inflammatory factor concentrations decrease, further demonstrating the positive impact of yeast peptides on the health of suckling calves. The nuclear factor (NF)-κB pathway is involved in the transcription of various pro-inflammatory cytokines, such as IL-6 and IL-8. It has been reported that selenium-enriched yeast peptides can inhibit NF-κB activation, reducing the levels of pro-inflammatory cytokines in a psoriasis-like dermatitis model (14). The p38 mitogen-activated protein kinase (MAPK) pathway is an important inflammatory mediator involved in the production of inflammatory cytokines. Yeast peptides also inhibit p38 MAPK phosphorylation, thereby alleviating inflammation (33). Although yeast peptides can improve the anti-inflammatory capacity of calves, the underlying mechanisms behind their anti-inflammatory activity require further validation.

Antioxidant capacity involves various mechanisms to resist and neutralize free radicals and other oxidative substances. Free radicals, which are molecules with one or more unpaired electrons, are highly reactive and can cause cellular damage, leading to disease. Although yeast peptide supplementation had no significant effects on the concentrations of enzymatic antioxidants (SOD, glutathione peroxidase, CAT, and MAD) in calf serum, the T-AOC in the AP group was significantly lower than that in the control group at 28 days of age. Typically, the high-risk period for calf diarrhea is from 0 to 28 days. Excessive oxidative stress may cause diarrhea and reduced immunity, which can lead to inflammation. From 28 to 56 days, as immunity increases, calves generally enter a stable growth phase. Considering the fewer diarrhea incidents in the AP calves, it is believed that the CON calves may have experienced more oxidative stress from 0 to 28 days, as they overexpressed antioxidants to counteract oxidative stress damage. This also explains why the antioxidant and inflammation-related indicators in the AP group calves showed significant changes at 28 days after birth but no significant differences at 56 days. Interestingly, we also found an interaction effect between treatment and age on T-AOC in the yeast peptide calves. Studies in animals and humans have shown that higher feed intake increases the metabolic burden, as digesting and metabolizing more food requires more energy, leading to increased production of free radicals and reactive oxygen species (ROS), thereby increasing oxidative stress. Combined with the significantly higher solid feed dry matter intake observed in the AP group calves compared to the CON group, we believe that AP affected antioxidant capacity, possibly involving the combined effects of initial stress and subsequent adaptive responses in calves.

## 5 Conclusions

This study demonstrates that yeast peptide supplementation during the suckling period can reduce the duration of diarrhea and the incidence of diarrhea in calves. Additionally, daily supplementation with 5 g of yeast peptides greatly improved the serum antioxidant capacity and anti-inflammatory and immune functions in calves. Future studies should explore the optimal dosage and mechanisms behind the anti-inflammatory activity of yeast peptides.

## Data Availability

The original contributions presented in the study are included in the article/supplementary material, further inquiries can be directed to the corresponding author.
